# PADP: progressive and adaptive data pruning for efficient incremental learning

**DOI:** 10.1038/s41598-026-43959-x

**Published:** 2026-03-13

**Authors:** Biqing Duan, Di Liu, Zhenli He, Wei Zhou, Shengfa Miao

**Affiliations:** 1 School of Software, Yunnan University, Kunming, China; 2 Department of Computer Science, NTNU, Trondheim, Norway

**Keywords:** Engineering, Mathematics and computing

## Abstract

Data pruning is a key technique for reducing training costs and improving model performance. However, most existing methods rely on fixed pruning rates or single metrics, which are largely designed for static training settings, making them unsuitable for incremental learning, where data distributions and model states change dynamically. To address this, we propose PADP, a progressive and adaptive data pruning method for incremental learning, which dynamically evaluates sample difficulty and its difficulty changes during training to enable adaptive sample selection for each incremental learning task. Specifically, we propose two metrics, the instant difficulty score and the difficulty variation score. The former evaluates the learning difficulty of a sample, while the latter evaluates the variation in difficulty over a training interval. These two metrics are combined to guide pruning decisions. To prevent certain classes from being completely removed, we also introduce a class-balance retention mechanism. Experimental results show that PADP outperforms existing data selection methods on CIFAR-100 and Tiny-ImageNet, generalizes across multiple incremental learning frameworks, and still maintains or exceeds the original accuracy even when training time is reduced by up to 52.90% compared to using the full dataset, demonstrating its effectiveness and practical value.

## Introduction

Deep learning has achieved remarkable progress in fields such as computer vision and natural language processing, driven by the continuous expansion of model scale and the availability of massive datasets^1^. However, the ever-growing size of models and data has also led to substantial computational, storage, and energy burdens^2,3^. The high resource consumption and infrastructure costs required for training large-scale models have become a major obstacle to their broader deployment^4^. To address this issue, researchers have proposed various approaches to reduce training costs, including parallel computing^5,6^ and model compression^7,8^. Recently, data-efficient approaches, which select or prune training data to remove redundant samples and unnecessary computation, have gained increasing attention. These approaches can be directly applied to different models without modifying their architectures. They not only reduce resource consumption and training cost but also improve model accuracy^9^.

Existing data-efficient methods can be generally categorized into two groups. The first is core-set selection^10^, which aims to identify a representative subset of the full dataset for efficient training. These methods typically evaluate sample importance based on geometric distribution^11,12^, sample diversity^13,14^, or gradient similarity^15,16^. The second group is data pruning^9^, which dynamically removes redundant or low-value samples during training or at specific training stages. Such methods usually estimate sample importance according to *uncertainty*^17,18^, *training dynamics*^19,20^, *influence functions*^21,22^, or *a combination of multiple criteria*^23,24^, thereby effectively reducing computational cost and accelerating training.

Although core-set selection and data pruning methods have demonstrated good efficiency and performance in traditional classification tasks, they are primarily designed for static learning rather than incremental learning (IL)^25^. IL features an online learning paradigm in which models continually acquire new knowledge as data for new classes becomes available after deployment^26^. The main challenge is retaining previously learned knowledge while continually incorporating new classes, thereby mitigating the well-known problem of *catastrophic forgetting*. As an increasing number of models have been implemented on resource-constrained devices with unknown tasks, these systems are increasingly expected to learn the new tasks at runtime or on the device. Therefore, *data-efficient IL* methods are expected.

In contrast to traditional static learning, where all data can be accessed and trained jointly, IL operates on a continuously evolving data stream. In IL, training samples not only change as training progresses, but also varies significantly across different incremental phases. Applying data pruning to IL poses new challenges, i.e., data pruning must not only reduce training cost but also ensure to mitigate *catastrophic forgetting* for the model. Existing data pruning approaches exhibit notable limitations in this regard. (1) Most methods rely on a one-time evaluation of the samples to select which data to retain or prune, failing to capture dynamic changes in the IL training^27,28^. In IL, one-time evaluation and then pruning is unreliable: *over-pruning risks removing samples that become important in later stages and degrading IL performance, while under-pruning limits the reduction in training cost.* Therefore, a progressive pruning strategy is thus needed. (2) Many approaches adopt a fixed-ratio data pruning, that is not suitable for IL. The learning difficulty of each incremental task varies, and a fixed pruning ratio may not suit all tasks. Therefore, an adaptive strategy that can flexibly adjust the number of pruning samples according to the data and characteristics of each incremental task is desired. (3) In addition, to mitigate catastrophic forgetting, many IL methods retain a small number of old class samples or prototypes to train alongside new class samples. Therefore, data pruning must ensure that both old and new class samples are effectively preserved, preventing categories with initially few samples from being entirely lost during later pruning stages. An effective data pruning strategy in IL needs to take this into consideration.

To address these limitations, we propose a progressive and adaptive data pruning method (PADP) designated for IL. In each incremental phase, PADP dynamically evaluates the importance and uncertainty of samples through the Instant Difficulty Score (IDS) and the Variation of Difficulty Score (VDS), enabling adaptive pruning with a non-fixed pruning ratio. IDS quantifies the instantaneous learning difficulty of a sample by measuring the discrepancy between the model’s prediction and the true label, while VDS captures the evolution of a sample’s difficulty throughout training to identify “*real*” low-value samples. Based on a combined score of these two metrics, PADP performs progressive pruning in multiple steps within each incremental phase, gradually removing low-value samples to avoid prematurely discarding critical ones while significantly reducing training costs. Additionally, to avoid the class imbalance caused by data pruning during each incremental phase, we incorporate a class-balanced retention mechanism to preserve class diversity, preventing performance degradation due to excessive loss of minority-class samples.

The main contributions of this work are summarized as follows:

1. We propose a sample scoring method that combines IDS and VDS. This mechanism considers both the instantaneous learning difficulty of samples and their dynamic changes during training, providing a more accurate reflection of sample importance and stability in IL.
2. A progressive and adaptive pruning strategy is developed, which gradually prunes samples in multiple steps within each incremental phase and dynamically determines the samples to be removed based on the scoring results. A class-balanced retention mechanism is incorporated to maintain the diversity and fairness of the training data.
3. Experimental results on CIFAR-100 and Tiny-ImageNet datasets demonstrate that PADP reduces training time by up to 52.90% while achieving a 6.45% improvement in accuracy compared with training on the full dataset, highlighting its efficiency and effectiveness.

## Related work

### Incremental learning

IL differs from the traditional static learning paradigm, in which a model is trained once on a full dataset before deployment. The goal of IL is to continuously learn new classes on top of a pretrained model, thereby enabling the progressive accumulation of knowledge. However, IL faces the challenge of *catastrophic forgetting*, that is, the prediction accuracy of old classes drops as the model learns new classes. Early methods alleviate forgetting through regularization. EWC^29^ constrains critical weights based on parameter importance, while LwF^30^ employs knowledge distillation to preserve knowledge of previous tasks. However, regularization-based IL methods rely on the evaluation of parameter importance, but parameter importance can dynamically change across different incremental stages, making it difficult to balance old knowledge and new knowledge. Replay-based approaches reuse a small subset of old data during training, allowing the model to better retain knowledge of previous classes. iCaRL^31^ is the first to combine knowledge distillation with sample replay to maintain representations of old classes, and PODNet^32^ and DER^33^ further improve feature distillation, helping the model retain reliable performance on old classes while learning new ones. Although replay effectively mitigates forgetting, its performance highly depends on how representative samples are selected and stored, especially under limited memory budgets. In comparison, prototype-based methods^34,35^ leverage class centroids for feature transfer, reducing storage and computation costs while maintaining representation consistency. Moreover, dynamic architecture-based methods^36,37^ adapt the model structure to new classes, further improving flexibility, but often introduce additional parameters that may hinder deployment on resource-limited devices. Recently, the development of pre-trained models^38,39^ has demonstrated that feature consistency and transferability are crucial for IL performance. LODAP^40^ strive to use an efficient model structure with a simple data pruning approach to conduct on-device IL, but their method does not take into account of the dynamic change of learning difficulty of each sample. Applying data-efficient techniques to IL, such as data pruning, has not been widely explored.

### Data pruning and core-set selection

The large amount of data is widely considered to be one of the key factors contributing to recent breakthroughs in artificial intelligence. However, researchers have begun to question whether all data in a dataset are equally important for training a model. Data pruning , core-set selection and data augmentation are important techniques for data-efficient learning and model training optimization, aiming to select a compact and representative subset of samples from the full dataset to reduce training cost while improving model performance. Static data pruning methods typically rely on a single criterion for sample selection. For example, Forgetting^19^ quantifies a forgetting score by tracking how frequently a sample changes from correct to incorrect predictions to guide sample selection.^22,41^ use influence functions to estimate the impact of removing each sample on model performance, thereby identifying redundant data. GraNd^9^ measures sample difficulty based on gradient norm to prune data, while EL2N^9^ evaluates sample difficulty in the early stage of training using the norm of the error vector. CRAIG^15^ constructs a small core-set through weighted greedy sampling and gradient matching, allowing model training on the core-set to replace full dataset training.

Dynamic data pruning methods often leverage multi-dimensional criteria to adaptively guide sample selection. For instance,^42^ selects samples based on uncertainty and novelty. BOSS^43^ balances feature similarity and label variability to achieve size-aware one-shot subset selection. YOCO^28^ dynamically adjusts the dataset size using two rules based on prediction error and class balance.^44^ combines reinforcement learning with the model’s real-time training state for sample selection.^18^ identifies and removes samples with low contribution to model performance by dynamically evaluating prediction uncertainty during training. DynImpt^24^ uses a combined score of sample loss, instability, and inconsistency for dynamic sample selection.

Although the methods have achieved significant improvements in training efficiency and model performance, most of them are primarily designed for static learning scenarios and do not explicitly account for the staged and progressive nature of IL. Unlike one-shot pruning on a fixed dataset, IL requires sample selection to be performed across sequential tasks, where both the data distribution and the model parameters constantly evolve. Consequently, static pruning strategies struggle to preserve representative samples across tasks and often amplify catastrophic forgetting. Our method is specifically designed for the incremental learning setting, where both the data distribution and model parameters evolve across stages. Instead of applying one-shot pruning on a fixed dataset, it performs progressive sample selection throughout the incremental process, better aligning the pruning strategy with the staged and evolving nature of IL.

## The progressive and adaptive data pruning

**Fig. 1:**
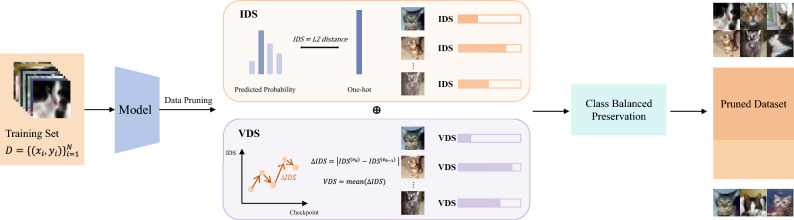
The overview of PADP.

In this section, we introduce PADP, a progressive and adaptive data pruning framework designed for IL, which incorporates both sample importance evaluation and a progressive pruning strategy, rather than being a standalone sample importance evaluation method. The overview of PADP is shown in Fig. 1. The core of PADP is two scores: IDS and VDS, used to evaluate the importance of data and to help prune unimportant data in each incremental task. IDS measures the learning difficulty of a sample at the current epoch, while VDS captures the dynamic variation in sample difficulty throughout training. The following section provides detailed definitions of both metrics and explains the rationale behind their design. Before delving into the details, we first proceed to the formal problem definition.

### Problem definition

We consider a standard IL setting^31^, where a model is trained on *n* sequential tasks $\mathscr {T} = \left\{ T_{0}, \ldots , T_{n-1} \right\}$. Each task $T_{t}$ in the *t*-th step introduces a set of new classes $\mathscr {Y}_t$ that are previously unseen. The training dataset for task $T_{t}$ is denoted as $\mathscr {D}_t = (x_i, y_i)_{i=1}^{N}$, where $x_i \in \mathbb {R}^d$ is an input sample and $y_i \in \mathscr {Y}_t$ is its corresponding label. At each task $T_{t}$, the model is trained only on the current dataset $\mathscr {D}_t$(which may include old-class samples for forgetting mitigation, depending on the IL paradigm), but performs unified classification over all seen classes in $\bigcup _{k=0}^{t} \mathscr {Y}_k$. This setting is designed to simulate real-world scenarios where models continuously receive new class information.

As shown in previous work^9,19^, some samples in the training set provide more valuable knowledge or information for a model to improve its accuracy. On this basis, our goal is to dynamically select the most valuable training samples for each incremental task $T_t$ to* improve accuracy and mitigate catastrophic forgetting while reducing training cost*. **This will benefit the emerging trend of on-device machine learning**^45^.

### The proposed approach

The critical part of data pruning is to determine which sample in a dataset is more important and contributes more to a model accuracy

#### Instant difficulty score

Since the information in training samples varies, the difficulty of learning different samples also differs during training. Inspired by EL2N^9^, sample difficulty is used as an evaluation metric to choose samples that contribute more to model generalization effectively. The IDS measures the learning difficulty of each sample by quantifying the discrepancy between the model’s predicted probability vector and the corresponding ground-truth label. *“instant”* represents the model’s mastery of the sample in the current epoch in an IL task. Samples with higher IDS indicate that the model has not yet fully learned their features at the current stage, and retaining such samples can help improve the model’s generalization performance, especially under the constraints of limited computing and storage resources in IL. Conversely, samples with lower IDS scores suggest that the model has already learned their feature information, making it potentially redundant for subsequent model updates. Removing these easy samples helps reduce computational overhead without significant loss of performance. Therefore, IDS can be an effective criterion for data selection, guiding pruning strategies to focus on retaining samples that are currently more informative and challenging.

Let $e\in \mathscr {E}$ denote a pruning point when a data pruning is conducted in an IL task $T_t$, i.e., a specific training epoch, where $\mathscr {E}$ denotes all pruning points for the IL task. We define the model at *e* as $f_{\theta ^{(e)}}$, where $\theta ^{(e)}$ represents the model parameters at that pruning point. Given a training sample $(x_i, y_i) \in \mathscr {D}_t^{(e)}$, the model outputs a logit vector $f_{\theta ^{(e)}}(x_i) \in \mathbb {R}^C$, where *C* represents the number of training data classes in task $T_t$. The prediction is then transformed into a probability distribution via the softmax function:

1 $$\begin{aligned} {\bf p}_i^{(e)} = \textrm{softmax}(f_{\theta ^{(e)}}(x_i)), \end{aligned}$$

where ${\bf p}_{i}^{(e)} \in \Delta ^{C-1}$ denotes the predicted probability vector at epoch *e*.

Let ${\bf e}_{y_i} \in \{0,1\}^C$ denote the one-hot encoding of the true label $y_i$. The IDS at epoch *e* for sample $x_i$ is defined as:

2 $$\begin{aligned} \textrm{IDS}_i^{(e)} = \left\| {\bf p}_i^{(e)} - {\bf e}_{y_i} \right\| _2, \end{aligned}$$

this L2-norm corresponds to the Euclidean distance between the predicted class probabilities and the true label, reflecting how well the model fits the sample. The IDS values are calculated for all samples in $\mathscr {D}_t^{(e)}$. From a computational complexity perspective, the computation of IDS is based on the predicted probabilities obtained during the standard forward pass and does not require additional backpropagation or auxiliary networks, resulting in relatively low computational overhead.

IDS can accurately reflect the model’s current mastery of each training sample, distinguishing between difficult samples that have not been sufficiently learned and easy samples that the model has well understood. This metric mainly captures the static difficulty of the sample at a training epoch. As one of the metrics for sample selection, it can guide the model to prioritize the samples that contribute significantly to model performance, thereby effectively reducing training computation while maintaining the model accuracy.

#### Variation of difficulty score

During model training, the model’s parameters are continually updated, causing its predictions for the same sample to vary over time. Although IDS reflects the model’s static learning states for the sample at a specific moment, this score itself fluctuates throughout training. Therefore, the difficulty score of a sample at a specific point in time is insufficient to comprehensively characterize whether the sample remains valuable for model learning throughout the entire training period, especially in IL, where the model may exhibit forgetting for some samples later because of mixing the features of old and new classes. To address this, we propose the *Variation of Difficulty Score* (VDS), which quantifies how a sample’s IDS fluctuates across training epochs. A high VDS indicates that the model’s learning of the sample is unstable and that the sample requires more attention for the model to capture its more features. By capturing these temporal fluctuations, VDS highlights samples whose learning is unstable, enhancing the robustness of the overall scoring mechanism. Compared to a one-time evaluation, VDS provides a more comprehensive assessment of sample importance, which is especially critical in IL where the model may forget previously learned samples.

Let $\mathscr {E} = \{e_1, e_2, \ldots , e_K\}$ denote the ordered set of *K* pruning points, i.e., certain epochs in a IL training. At the current pruning point $e_k \in \mathscr {E}$ where $k \ge 2$, we compute the VDS for a training sample $(x_i, y_i)$ based on its historical IDS values from all preceding pruning points up to $e_k$. Specifically, the score sequence is:

3 $$\begin{aligned} \mathscr {S}_i^{(k)} = \left\{ \textrm{IDS}_i^{(e_1)}, \textrm{IDS}_i^{(e_2)}, \ldots , \textrm{IDS}_i^{(e_k)} \right\} . \end{aligned}$$

We compute the average change in $\textrm{IDS}$ values between adjacent pruning points to measure the model’s changing assessment of the sample over time. Specifically, at the current pruning point $e_k$, the VDS for sample $x_i$ is defined as:

4 $$\begin{aligned} \textrm{VDS}_i^{(e_k)} = \frac{1}{k - 1} \sum _{j = 2}^{k} \left| \textrm{IDS}_i^{(e_j)} - \textrm{IDS}_i^{(e_{j-1})} \right| , \end{aligned}$$

Note that the VDS of the first pruning point is set to 0.

This score captures the stability of the model learning a specific sample. A higher VDS indicates that the model’s understanding of the sample is unstable, possibly due to occasional forgetting during the training process. In contrast, a lower VDS suggests a more stable learning behavior, where the model either continuously learns the sample or persistently ignores it. Noteably, the computation of VDS only involves simple numerical differences of previously computed IDS values across pruning checkpoints, and does not require additional model inference or gradient computation.

Compared to IDS, which provides sample difficulty at a single point in time, VDS incorporates temporal dynamics, enabling a more comprehensive evaluation of a sample’s reliability and learning contribution. As such, VDS serves as a complementary indicator to IDS, enhancing the robustness and discriminative capacity of the final sample scoring and providing a more solid foundation for pruning decisions.

#### Class-balanced retention mechanism

The combination of IDS and VDS can be used to identify low-contributing data in each IL task, but this method is class-agnostic, meaning it may remove many or all samples from certain classes. *However, IL requires preserving samples from previously learned classes, either for replaying them or for generating prototypes. Otherwise, classes without samples are likely to be forgotten as more classes are learned.* To ensure the diversity and fairness of data in the pruned dataset, we design a simple yet effective class-balanced retention mechanism. Before proceeding to the approach, we first introduce the metric combining IDS and VDS. After obtaining the evaluation scores from two different perspectives, we calculate a unified difficulty score to comprehensively evaluate the importance of each sample during the training process. The fused score serves as a basis for guiding subsequent data pruning. Since the IDS and VDS scores differ in scale and distribution, we apply normalization to ensure they contribute proportionally when fused. This normalization eliminates the scale differences between different indicators, making the two types of scores comparable at the same statistical scale.

The final fused score $F_i$ for sample $x_i$ is calculated as a weighted sum of the normalized IDS and VDS:

5 $$\begin{aligned} F_i = \alpha \cdot \hat{\textrm{IDS}}_i + (1 - \alpha ) \cdot \hat{\textrm{VDS}}_i, \end{aligned}$$

where $\alpha \in [0,1]$ is a hyperparameter that controls the weight of each score in the final score, and $\alpha$ can be adjusted based on the relative importance between model training stability and difficulty in different tasks.The fused score $F_i$ serves as the final criterion for ranking and selecting samples in the pruning stage. The linear combination is adopted for its balanced integration of IDS and VDS after normalization. Compared with nonlinear operators such as geometric or harmonic means, linear weighting avoids excessive sensitivity to low values and provides a stable unified score for progressive pruning.

The class-balance retention approach is as follows. Before performing a pruning, we first retain the top *K* highest-scoring samples within each class:

6 $$\begin{aligned} \mathscr {D}_{\textrm{top}}^{(t,e)} = \bigcup _{c \in \mathscr {C}} \operatorname {Top}_K \Big ( \big \{ (x_i, y_i) \in \mathscr {D}_c^{(t,e)} \big \} \text { sorted by } F_i \Big ), \end{aligned}$$

For each pruning point $e \in \mathscr {E}$ in the incremental task $T_t$, the number of samples to be kept is defined as:

7 $$\begin{aligned} N_{\text {keep}}^{(t,e)} = (1-\gamma ^{(e)})|\mathscr {D}_t^{(e)}|,\quad \gamma ^{(e)} = \gamma _{\text {start}} + \left( \frac{e}{E} \right) \cdot (1 - \gamma _{\text {start}}), \end{aligned}$$

where $|\mathscr {D}_t^{(e)}|$ denotes the size of the training set before pruning at pruning point *e*, and $\gamma ^{(e)}$ represents the pruning ratio, which starts from the initial pruning ratio $\gamma _{\text {start}}$ and increases linearly as training progresses, gradually pruning the dataset. *E* denotes the total number of training epochs for task $T_t$, while $\frac{e}{E}$ calculates a linear coefficient to increase the pruning ratio. Subsequently, among the remaining samples, we select the rest of the samples to keep according to their $F_i$ scores.

8 $$\begin{aligned} \mathscr {D}_{\text {remain}}^{(t,e)} = \operatorname {Top}_{N_{\text {keep}}^{(t,e)} - \left| \mathscr {D}_{\text {top}}^{(t,e)}\right| } \left( \mathscr {D}_t^{(e)} \setminus \mathscr {D}_{\text {top}}^{(t,e)} \ \text {sorted by} \ F_i \right) , \end{aligned}$$

the subset after pruning point *e* is defined as:

9 $$\begin{aligned} \mathscr {D}_{\text {pruned}}^{(t,e)} = \mathscr {D}_{\text {top}}^{(t,e)} \cup \mathscr {D}_{\text {remain}}^{(t,e)}. \end{aligned}$$

This mechanism ensures that each class retains at least *K* samples, preventing any class from completely losing its samples and effectively mitigating the adverse effects.

#### The algorithm

With IDS, VDS, and the class-balance retention method, we present the details of PADP. PADP adopts a progressive and adaptive pruning strategy that removes data step by step, fully accounting for the dynamic changes in sample importance as the model evolves. The approach starts to prune the dataset after an initialization stage that trains the model to learn sufficient feature representations of new classes in the IL task.

The pseudocode of PADP is shown in Algorithm 1. At the initial stage of training, the model is trained on the complete dataset until the first pruning point is reached. At each subsequent pruning point epoch $e \in \mathscr {E}$, we compute the fused difficulty score $F_i$ for all current samples. We adopt a progressive and adaptive pruning strategy to determine the number of samples to be pruned in each round. Unlike methods that predefine a fixed pruning ratio, we gradually increase the pruning ratio as the training progresses, allowing the pruning process to adapt to the evolving representation capability of the model.

After determining the number of samples to prune by $\gamma ^{(e)}$, we sort all samples based on their $F_i$ scores, and prune the samples according to the class-balanced retention mechanism. The pruned subset $\mathscr {D}^{(t,e)}_{\text {pruned}}$ is then used as the training data for the current phase and remains unchanged until the next pruning point is triggered. Except for the first pruning, each subsequent pruning operation is conducted based on the dataset retained after the previous pruning, allowing the selected sample subset to dynamically adapt to the evolving state of the model during training.

**Algorithm 1:**
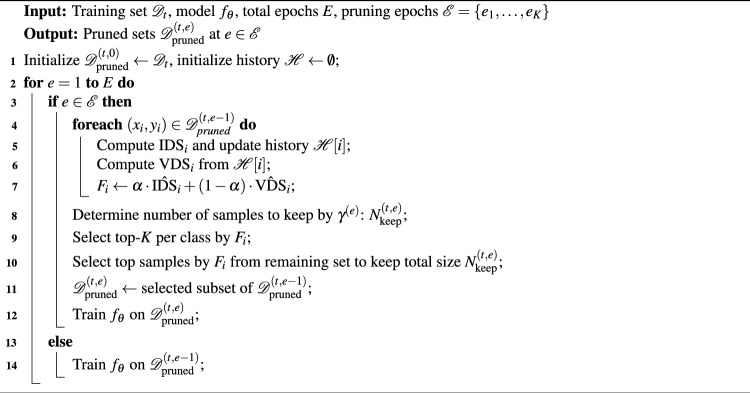
Progressive and adaptive data pruning.

## Experiments

### Dataset and experimental settings

#### Datasets and partition protocols

To evaluate the effectiveness of our method, we conduct experiments on the two most commonly used datasets in IL. The details of the datasets are as follows:

CIFAR-100^46^ is a medium-scale image dataset consisting of 100 classes and a total of 60,000 images. Each class contains 600 images, including 500 training images and 100 testing images. The image resolution is $32 \times 32$ pixels. Tiny-ImageNet^47^ is a subset of ImageNet-1k, containing 200 classes and a total of 120,000 images. Each class contains 600 images, with 500 training images, 50 validation images, and 50 testing images. The image resolution is $64 \times 64$ pixels.

For the order and division of all dataset classes for IL in our experiment, we follow the setting in^35^. All dataset classes are arranged in a fixed random order. We divide IL into an initial phase and multiple incremental phases. The initial phase uses half of the dataset classes for training, and the remaining classes are evenly divided into *p* phases for subsequent incremental training.

#### Comparison approaches

We selected representative data pruning methods as comparison approaches to validate the effectiveness of the proposed PADP, such as Forgetting^19^, EL2N^9^, Entropy^27^, CCS^13^, Moderate-DS^48^, AUM^49^ and MoSo^50^.

- Forgetting^19^ evaluates the importance of samples by defining forgetting events. If a sample is correctly classified in one epoch but misclassified in subsequent epochs, it is considered to have experienced a forgetting event. Throughout the training process, samples with more accumulated forgetting events are generally regarded as more contributive to the model’s generalization ability and are therefore preferentially retained for constructing the training subset.
- EL2N^9^ measures the difficulty and informativeness of samples by calculating the Euclidean distance between the model’s predicted probabilities and the true labels during the early training phase.
- Entropy^27^ measures the uncertainty of a sample by computing its entropy value. A higher entropy indicates that the model’s prediction for the sample is more uncertain and that the sample contains more information, making it more likely to help improve the model during training.
- CCS^13^ selects a subset of samples that best represents the original data distribution by constructing coverage relationships in the feature space. It adopts a greedy strategy to optimize the coverage metric in the embedding space, prioritizing representative samples.
- Moderate-DS^48^ measures sample importance by computing the Euclidean distance between each sample and its class center, and selects samples with distances close to the median to construct the subset.
- AUM^49^ measures sample uncertainty by recording, during training, the margin between the logit score of the true label and the highest logit score among the incorrect labels for each sample, and then computing the average margin across all training epochs. A smaller average margin indicates more unstable model prediction on that sample, which can be prioritized for removal during subset selection.
- MoSo^50^ evaluates sample importance by estimating the change in optimal empirical risk when a sample is removed. To avoid costly retraining, it uses a first-order gradient-based approximation: samples whose gradients consistently align with the average gradient across training stages are deemed more representative and beneficial for optimization, thus assigned higher scores and retained preferentially.

#### Implementation details

All experiments are conducted on an Ubuntu 20.04 system using an NVIDIA GeForce RTX 4090 GPU, based on a Python 3.9 and PyTorch 1.12^51^ environment. We employ the iCaRL^31^ method for IL, with a ResNet-18^52^ backbone network. In Section 4.4, we also evaluate PADP across diverse IL methods. For data augmentation, random cropping, horizontal flipping, and normalization operations are applied to all training data. The optimizer used in all experiments is SGD, with a momentum coefficient of 0.9 and weight decay of 0.0005. For the IL training settings, the initial training phase consists of 200 epochs, with each incremental phase training for 170 epochs. The initial learning rate is 0.1, and a multi-step learning rate decay strategy is adopted, where the learning rate is multiplied by 0.2 at epochs 60, 120, and 160. The batch size is set to 128. In terms of data settings, the number of incremental stages for CIFAR-100 and Tiny-ImageNet is set to $p=5$ and $p=10$. All pruning operations are performed during the incremental stages.

In the comparison methods, the first 50 epochs are used as a training warm-up phase, after which a one-time sample pruning is conducted based on each model’s evaluation metric. The selected subset is then used to continue training for the remaining 120 epochs. This setting follows the common usage of these pruning methods in static learning, where a warm-up stage is required to obtain reliable sample evaluation before pruning. It also ensures a unified training protocol under the incremental learning setting without introducing a separate full retraining stage. *This setting is determined based on the setting deployed in their static learning environment.* Given that all comparison methods use one-time pruning, this setup highlights the overall differences between methods rather than comparing them solely based on sample evaluation metrics. All comparison methods perform one-time pruning under predefined pruning ratios, with pruning ratios set to 0.2, 0.4, 0.6, and 0.8.

For the comparison methods, we adopt a unified protocol in which the first 50 epochs are used as a training warm-up phase, after which a one-time sample pruning is conducted based on each model’s evaluation metric. The selected subset is then used to continue training for the remaining 120 epochs. *This setting follows the common usage of these pruning methods in static learning, where a warm-up stage is required to obtain reliable sample evaluation before pruning. * It also maintains a unified training protocol under the incremental learning setting without introducing an additional full retraining stage. Since all comparison methods follow their original one-time pruning design, this setup enables comparison of different sample evaluation metrics and pruning mechanisms under consistent training conditions. All comparison methods perform one-time pruning under predefined pruning ratios, with pruning ratios set to 0.2, 0.4, 0.6, and 0.8.

We present two alternatives for PADP:

1. PADP (default setting), which performs progressive pruning during training without pre-specifying a final target pruning ratio. Pruning is only conducted in the middle portion of the training process, specifically between 20% and 80% of the total training epochs, with pruning performed every 10% of the total epochs. We set pruning to start at epoch 34 and end at epoch 136 within the 170-epoch incremental training phase, with a pruning interval of 17 epochs. The score fusion weight is set to $\alpha = 0.7$, the initial pruning ratio to $\gamma _{\text {start}} = 0.2$, and $K = 20$ ensures that at least 20 samples per class are preserved to maintain class balance.
2. Fixed target pruning ratio (PADP-Fixed), which applies the same progressive pruning mechanism as the default setting, but requires a predefined target pruning ratio and prunes samples until the specified ratio is reached. Then, we could have a fair comparison between PADP and comparison approaches.

All primary experiments are independently conducted under three different random seeds, and the results are averaged to enhance robustness. The same seed configuration is applied to all comparison methods to ensure fairness and reproducibility.

### Main results

**Table 1:** The average accuracy (%) and standard deviation of different methods for CIFAR-100 and Tiny-ImageNet at different pruning ratios and incremental stages.

Method	Year	Prune ratio	CIFAR-100		Tiny-ImageNet	
			$p=5$	$p=10$	$p=5$	$p=10$
Random	-	0.20	44.61±0.17	44.08±0.21	19.81±0.22	13.42±0.18
		0.40	44.76±0.13	44.54±0.19	19.71±0.23	14.26±0.16
		0.60	44.52±0.25	41.38±0.20	19.03±0.19	14.89±0.26
		0.80	50.66±0.30	36.87±0.46	19.83±0.51	17.44±0.39
Forgetting^19^	2018	0.20	47.07±0.18	46.20±0.24	20.97±0.16	16.03±0.33
		0.40	43.56±0.22	48.61±0.29	20.44±0.19	17.38±0.37
		0.60	51.31±0.45	45.16±0.51	21.38±0.24	19.29±0.15
		0.80	52.04±0.33	39.07±0.28	24.81±0.40	18.91±0.32
EL2N^9^	2021	0.20	44.85±0.26	42.55±0.19	19.71±0.37	15.93±0.66
		0.40	44.23±0.25	45.64±0.31	19.40±0.62	15.58±0.58
		0.60	45.93±0.74	43.38±0.48	19.31±0.54	15.37±0.67
		0.80	50.10±1.30	38.31±0.33	19.18±0.51	17.68±0.79
Entropy^27^	2019	0.20	45.65±0.19	43.49±0.25	19.55±0.42	14.33±0.36
		0.40	44.84±0.31	44.02±0.29	19.17±0.44	13.96±0.38
		0.60	43.24±0.46	43.43±0.78	18.27±1.16	14.43±0.54
		0.80	50.45±0.48	36.48±0.73	19.50±0.81	17.96±1.25
CCS^13^	2022	0.20	45.41±0.15	42.32±0.42	20.19±0.37	15.07±0.28
		0.40	43.96±0.23	45.73±0.49	19.78±0.31	14.93±0.54
		0.60	48.66±0.71	42.83±0.64	20.18±1.26	15.84±1.30
		0.80	48.81±0.59	35.59±0.88	20.46±0.45	16.72±1.21
Moderate-DS^48^	2022	0.20	45.91±0.22	48.29±0.24	20.71±0.28	16.60±0.36
		0.40	46.52±0.50	48.94±0.41	20.26±1.34	17.08±1.02
		0.60	52.17±0.19	45.31±0.24	21.43±0.32	19.20±0.54
		0.80	52.23±0.18	37.26±0.33	24.31±0.57	19.30±0.81
AUM^49^	2020	0.20	42.99±0.13	43.83±0.31	20.29±0.47	14.80±0.86
		0.40	43.11±0.45	45.35±0.23	20.18±0.93	14.26±1.19
		0.60	47.76±0.42	42.75±0.28	20.15±0.15	15.75±0.34
		0.80	47.20±0.79	36.76±0.41	20.71±0.86	17.18±0.60
MoSo^50^	2023	0.20	45.42±0.26	44.11±0.14	20.09±0.42	15.14±0.31
		0.40	46.75±0.17	45.52±0.35	20.20±0.41	16.09±0.61
		0.60	46.98±0.23	42.25±0.42	20.11±0.53	16.51±0.49
		0.80	52.15±0.36	33.95±0.92	19.77±0.68	19.39±0.55
PADP-Fixed		0.20	60.20±0.18	53.63±0.23	31.57±0.36	25.37±0.24
		0.40	59.84±0.26	55.92±0.36	32.80±0.15	26.34±0.33
		0.60	59.95±0.25	55.94±0.10	33.55±0.52	28.10±0.20
		0.80	**60.63±0.13**	**59.04±0.29**	**36.35±0.60**	**31.15±0.88**
PADP		–	**60.70±0.14**	**59.13±0.29**	**37.80±0.38**	**31.80±0.16**

Table 1 shows the overall accuracy results of different data pruning methods on the CIFAR-100 and Tiny-ImageNet datasets under both 5-phase and 10-phase IL settings.

In the fixed pruning ratio setting, PADP-Fixed achieves higher accuracy under all pruning ratios. For example, when the pruning ratio is 0.8, PADP-Fixed improves accuracy by 8.40% compared to the best baseline method and still surpasses the full-data training by 4.11% on CIFAR-100 with the 5-phase setting, demonstrating that it preserves informative samples while removing redundant ones.

**Fig. 2:**
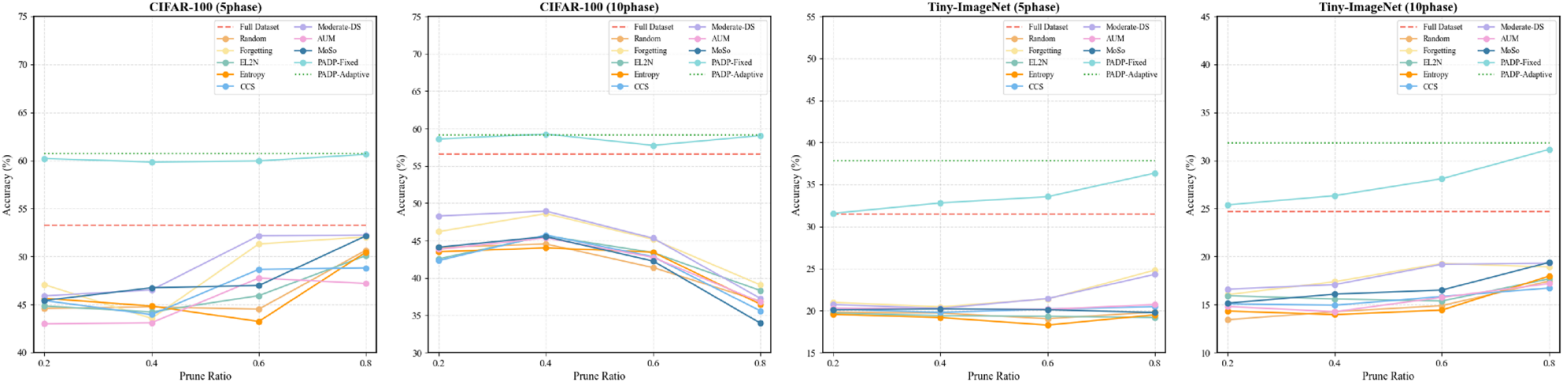
Illustrations of the average accuracy of all pruning methods for CIFAR-100 and Tiny-ImageNet at different incremental stages and pruning ratios.

In addition, PADP achieves comparable or even better performance without requiring a predefined pruning ratio. On Tiny-ImageNet with the 5-phase incremental setting, PADP achieves a 4.87% improvement over full-data training and further surpasses PADP-Fixed (0.8 pruning ratio) by 1.45%. These results verify that PADP can automatically adjust pruning strength based on the training dynamics and sample difficulty distribution, effectively balancing performance and efficiency during IL.

Figure 2 further illustrates the accuracy trends of different methods under various pruning ratios. As the pruning ratio increases, most baseline methods (e.g., EL2N^9^, CCS^13^) experience significant performance drops at high pruning levels, whereas PADP-Fixed maintains a consistently higher accuracy with smaller fluctuations. In contrast, PADP remains overall superior, showing that dynamic pruning effectively prevents over-pruning and performance degradation, thus achieving the best balance between stability and accuracy across different tasks and incremental stages. We observe that PADP sometimes achieves higher accuracy than training on the full dataset. This can be interpreted as an implicit regularization effect: progressive pruning removes redundant or noisy samples while preserving informative ones, which reduces noise and improves generalization. In addition, the class-balanced retention mechanism helps avoid performance degradation caused by class imbalance.

### Efficiency evaluation

In this subsection, we evaluate the computational efficiency of different data pruning methods in IL to analyze their practical feasibility. Experiments were conducted on CIFAR-100 and Tiny-ImageNet under a 10-phase incremental setting, with all methods employing a uniform pruning ratio of 0.8 to ensure a fair comparison of computational efficiency under high pruning ratio. Other experimental settings are consistent with those described in “Dataset and experimental settings”.

To provide a fair comparison of the number of samples pruned during the entire training process, Table 2 presents the sample retention ratios for PADP and one-shot fixed pruning ratio on the CIFAR-100 dataset throughout a single incremental training process. The “Avg.” indicates the weighted average sample retention ratio over all epochs, with weights corresponding to the number of training epochs in each phase, allowing for a fair comparison of overall pruning. The results show that the average sample retention ratio of PADP is close to that of the one-shot fixed pruning with a 0.8 pruning ratio. Table 3 reports the total training time of each method, along with the relative time reduction compared to the full dataset, computed as:

10 $$\begin{aligned} \text {Reduction} = \frac{T_{\text {full}} - T_{\text {pruned}}}{T_{\text {full}}}, \end{aligned}$$

where $T_\text {full}$ denotes the total training time using the complete dataset, and $T_\text {pruned}$ denotes the total training time after applying a pruning method.

From the results, the Random method achieves the highest time reduction on both datasets, as it directly selects samples from the training set without incurring additional sample-scoring overhead. However, this comes at the cost of a significant accuracy drop. Methods such as Forgetting^19^, EL2N^9^, and Moderate-DS^48^ require extra model inference or sample-level statistics during the pruning stage, slightly increasing training time compared to Random. Notably, CCS^13^ incurs higher total training time than even the full dataset on both datasets. This is primarily due to its core operations involving high-dimensional feature extraction and sample similarity matching, where complex coverage computations substantially increase computational overhead.

**Table 2:** Comparison of retained sample ratios between PADP and one-shot fixed pruning ratio.

Method	0–34	34–50	51–67	68–84	85–170	Avg. (%)
PADP	100	64.0	35.8	28.6	24.4	45.4
Fixed (0.8)	100	100	20.0	20.0	20.0	43.5

**Table 3:** Comparison of total training time(h) and reduction(%) ratio for CIFAR-100 and Tiny-ImageNet under the 10-phase setting.

Method	CIFAR-100		Tiny-ImageNet	
	Total time (h)	Reduction (%)	Total time (h)	Reduction (%)
Full Dataset	3.44	–	7.92	–
Random	1.44	58.14%	2.21	72.10%
Forgetting^19^	2.50	27.32%	2.21	72.10%
EL2N^9^	2.20	27.32%	3.11	60.73%
Entropy^27^	2.10	38.95%	2.96	62.62%
CCS^13^	3.88	-12.80%	9.99	-26.14%
Moderate-DS^48^	2.23	35.17%	2.99	62.25%
AUM^49^	2.15	37.50%	2.94	62.88%
MoSo^50^	2.59	24.71%	3.47	56.19%
PADP-Fixed	2.69	21.80%	3.63	54.16%
PADP	2.74	20.34%	3.73	52.90%

Our proposed PADP methods use a progressive pruning mechanism that dynamically performs sample scoring and data updates during training. As pruning proceeds, the number of samples gradually decreases, concurrently reducing the computational and storage burden of subsequent stages. PADP-Fixed achieves 21.80% and 54.16% training time reductions on CIFAR-100 and Tiny-ImageNet, respectively, while PADP achieves 20.34% and 52.90% reductions. Although the overall training time of PADP is slightly higher than that of PADP-Fixed, the difference is minimal. This is primarily due to the more conservative data pruning strategy during the early pruning stages, which slightly increases computation in those stages. This small overhead, however, improves accuracy, allowing PADP to consistently achieve superior performance across different datasets and incremental tasks.

### Evaluation on IL frameworks

In this subsection, we further evaluate the generality and adaptability of PADP across different IL frameworks. We adopt PADP and select five representative frameworks, including the prototype-based LODAP^40^, PASS^35^, and SSRE^34^, as well as the replay-based iCaRL^31^ and MEMO^37^. Experiments are performed on CIFAR-100 and Tiny-ImageNet with 10-phase and 5-phase IL tasks, respectively. Using the same network architecture and training settings as in “Dataset and experimental settings”, we compare two training processes: “without pruning” and “with pruning.”

**Table 4:** Comparison of accuracy (%) and forgetting (%) of different IL frameworks with and without pruning.

Method	CIFAR-100 (10 phase)				CIFAR-100 (5 phase)				Tiny-ImageNet (10 phase)				Tiny-ImageNet (5 phase)			
	Accuracy(%)		Forgetting(%)		Accuracy(%)		Forgetting(%)		Accuracy(%)		Forgetting(%)		Accuracy(%)		Forgetting(%)	
	w/o	w/	w/o	w/	w/o	w/	w/o	w/	w/o	w/	w/o	w/	w/o	w/	w/o	w/
iCaRL-CNN^31^	53.23	59.13	46.28	25.96	56.52	60.70	40.91	22.76	24.70	31.80	84.56	65.95	31.48	37.80	66.33	56.52
iCaRL-NCM^31^	53.76	57.05	29.26	25.04	57.16	60.06	27.12	18.16	33.53	34.19	64.08	63.10	38.28	40.85	50.30	44.75
PASS^35^	61.01	61.12	28.05	26.74	63.47	65.02	19.28	17.17	47.58	47.64	21.89	20.73	49.10	49.27	25.89	23.86
SSRE^34^	61.51	61.94	23.57	22.00	63.30	63.51	24.42	23.11	47.27	48.12	30.50	30.13	48.04	49.17	29.47	28.32
MEMO^37^	63.57	63.75	17.22	11.67	66.01	67.65	12.52	8.42	51.47	51.82	6.31	5.95	51.52	51.68	7.42	6.03
LODAP^40^	65.97	66.18	8.34	7.38	67.21	67.25	8.17	7.36	51.66	51.74	5.56	5.15	52.34	52.40	7.22	6.42

Table 4 reports the average accuracy and forgetting rate for each framework under different datasets and incremental settings. The forgetting rate is defined as the average decrease in performance of previous tasks relative to their highest achieved accuracy after completing IL, reflecting the model’s ability to retain learned knowledge. The results show that incorporating PADP generally leads to higher accuracy and lower forgetting rates across all frameworks. In the 10-phase CIFAR-100 setting, the average accuracy improves by up to 3.9% and the forgetting rate decreases by up to 20.32%. For the 10-phase Tiny-ImageNet tasks, the performance gain is even more noticeable, with some frameworks showing an average accuracy improvement of 7.1%. *Overall,* PADP *is more beneficial for replay-based IL approaches than prototype-based IL approaches*. Although the accuracy improvement is limited for prototype-based approaches, data pruning still significantly reduces training overhead and can benefit many on-device IL method.

### Ablation study

This section conducts ablation studies to analyze the contribution of each key component in PADP. Specifically, we evaluate (1) the effectiveness of the sample scoring indicators, (2) the necessity of the class-balanced retention mechanism, and (3) the impact of alternative sample evaluation metrics under a unified progressive and adaptive pruning strategy.

#### Effectiveness of evaluation indicators

**Fig. 3:**
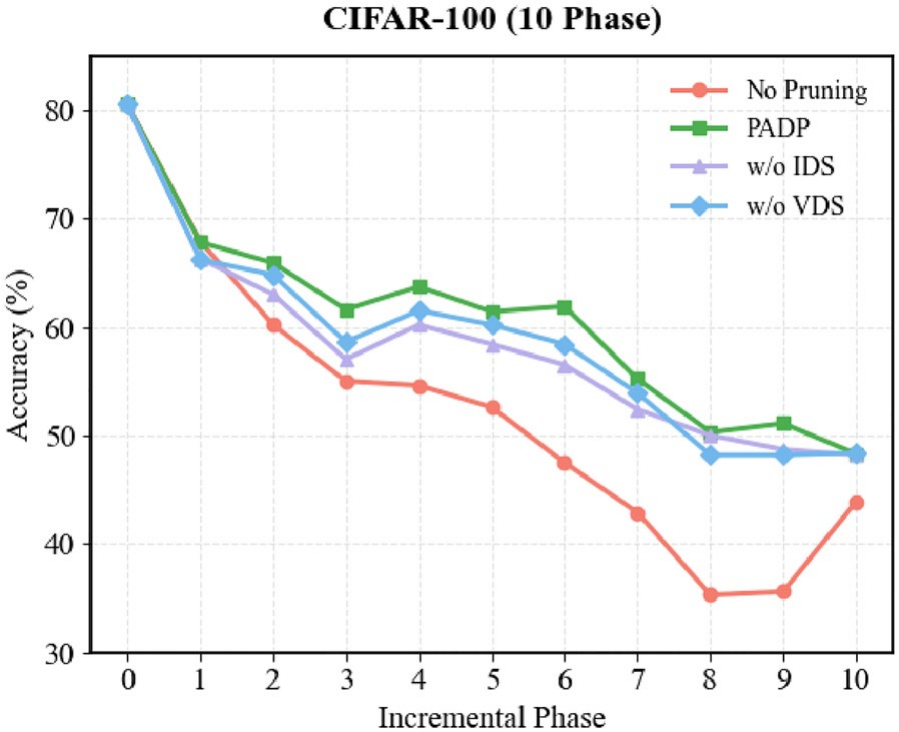
Ablation study on the effectiveness of evaluation indicators.

To validate the effectiveness of the proposed sample evaluation metrics, we conduct ablation experiments by removing either IDS or VDS from the scoring process. As shown in Fig. 3, we report the accuracy changes over 10 incremental phases on the CIFAR-100 dataset, while other configurations remain consistent with those described in “Dataset and experimental settings”. The results indicate that removing either score leads to a noticeable drop in model performance. Specifically, removing IDS results in a more pronounced accuracy decline during the early phases when new classes are introduced, suggesting that IDS helps to quickly identify critical samples and stabilize learning in the initial stages. In contrast, removing VDS causes greater performance degradation in the later phases, indicating that VDS can dynamically identify the most crucial samples for training at each phase based on the evolving sample difficulty, thereby optimizing sample selection and enhancing training continuity and stability.

Overall, the combined use of IDS and VDS yields more stable and superior performance. Compared with using either metric alone, PADP achieves an average accuracy improvement of approximately 2–3%. These results demonstrate the complementary roles of the two metrics: IDS focuses on selecting key samples in the early training stages to facilitate rapid adaptation to new classes, while VDS dynamically adjusts sample priorities in the later incremental phases, ensuring continuous and effective training, thereby achieving sustained performance improvement and mitigating catastrophic forgetting.

#### Effect of class-balanced retention mechanism

To further validate the effectiveness of the proposed class-balanced retention mechanism, this ablation study is conducted by varying the number of preserved samples, denoted as top *K*. The full PADP method is compared with a variant without this mechanism (i.e., $K=0$) as well as with different *K* values to evaluate both its effectiveness and sensitivity, using the CIFAR-100 dataset under the 10-phase incremental setting while keeping all other configurations as in “Dataset and experimental settings”. The results are summarized in Table 5, completely removing the class-balanced retention mechanism led to a significant performance drop, with the model achieving an average accuracy of only 45.83%. This is mainly because minority-class samples tend to be excessively reduced or even entirely removed after multiple pruning rounds, resulting in class imbalance. As *K* increases, the model accuracy improves noticeably, reaching 55.79% at $K=10$ and further increasing to 59.13% and 59.55% for $K=20$ and $K=50$, respectively, with the gains gradually tapering off. A larger *K* may reduce pruning flexibility and increase computational cost, therefore, $K=20$ is adopted as the default configuration, providing a favorable trade-off between accuracy and efficiency. Notably, $K=20$ is chosen based on the settings of CIFAR-100 and Tiny-ImageNet (each class containing 500 training samples), which retains approximately 4% of samples per class and balances class representativeness with pruning efficiency. In general, K can be adjusted proportionally to the per-class sample size of different datasets.

**Table 5:** Ablation study on the effect of the class-balanced retention mechanism with different top *K* values.

Top *K*	0	1	5	10	20	50
Accuracy (%)	45.83	47.57	52.16	55.79	59.13	59.55

These results demonstrate that the class-balanced retention mechanism effectively prevents dominant classes from overwhelming the pruning process, maintains the representativeness and diversity of the training subset, and thereby ensures more stable and sustained performance throughout IL.

#### Comparison with alternative evaluation metrics

To further assess the importance and effectiveness of the proposed dynamic sample scoring method for class-incremental learning, we conduct additional comparative experiments by combining alternative sample evaluation metrics with the same progressive and adaptive pruning strategy used in PADP.

Specifically, we replace the proposed IDS and VDS with the sample evaluation metrics from the comparison methods in “Comparison approaches”. For all compared methods, the progressive and adaptive pruning strategy is kept strictly identical, including the same pruning checkpoints, pruning frequency, and class-balance retention mechanism. The experiments are conducted on CIFAR-100 and Tiny-ImageNet under both 5-phase and 10-phase incremental learning settings. The overall results are reported in Table 6.

**Table 6:** Accuracy comparison of different evaluation metrics under the same pruning strategy.

Evaluation metric	CIFAR-100		Tiny-ImageNet	
	$p=5$	$p=10$	$p=5$	$p=10$
Random	53.92	53.66	21.74	17.95
Forgetting^19^	53.83	53.49	21.94	22.32
EL2N^9^	52.94	53.62	21.37	21.78
Entropy^27^	52.83	53.47	21.37	17.51
CCS^13^	53.14	53.23	21.44	17.54
Moderate-DS^48^	54.26	53.64	22.96	18.52
AUM^49^	53.29	54.15	22.66	19.29
MoSo^50^	53.12	53.01	21.62	17.56
PADP	60.70	59.13	37.80	31.80

As shown in Table 6, incorporating alternative sample evaluation metrics into the proposed progressive and adaptive pruning strategy yields certain performance improvements compared to their original pruning method, none of them consistently match or surpass the performance achieved by PADP.

As shown in Table 6, together with the main results presented in “Main results”, integrating the sample evaluation metrics of alternative methods with the proposed progressive and adaptive pruning strategy can yield certain performance improvements compared to their original pruning schemes. In class-incremental learning scenarios, the proposed IDS and VDS jointly consider both the instantaneous learning difficulty of samples and its dynamic changes during training, providing more informative guidance for data pruning and thereby achieving more superior overall performance. It should also be noted that the effectiveness of PADP depends on the coordinated combination of sample evaluation metrics and the progressive and adaptive pruning strategy, and there remains room for further improvement of the overall approach.

### Parameter sensitivity analysis

To further examine the robustness of PADP with respect to key hyperparameters, we conduct the sensitivity analyses on the score fusion weight $\alpha$ and the initial pruning ratio $\gamma _{\text {start}}$.

#### The score fusion weight $\alpha$

In PADP, the final sample score is obtained by linearly combining the static difficulty metric IDS and the dynamic variation metric VDS, where $\alpha$ controls the contribution of IDS. To evaluate the effect of different weight settings, we conduct experiments on the CIFAR-100 dataset with $p=10$ incremental phases, while keeping all other experimental configurations unchanged. The value of $\alpha$ is varied from 0.1 to 1.0.

**Fig. 4:**
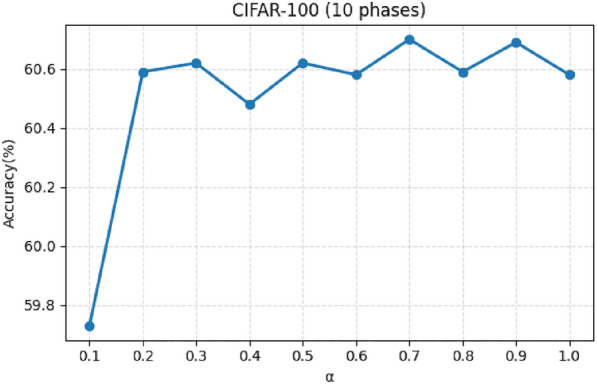
Accuracy under different value of $\alpha$.

The results are shown in Fig. 4. It can be observed that the model accuracy remains relatively stable under different weight settings, with the accuracy consistently above 59% and a maximum variation of approximately 0.97 percentage points. This indicates that PADP is not highly sensitive to the precise choice of $\alpha$ and can achieve competitive performance without careful tuning. Further observation shows that the performance reaches its best around $\alpha = 0.7$, which supports our default configuration. Meanwhile, using only static scoring or only dynamic scoring does not achieve optimal performance, which further supports the complementary relationship between IDS and VDS. Overall, these results demonstrate that PADP maintains stable performance within a relatively wide range of $\alpha$, reflecting good robustness in practical applications.

#### The initial pruning ratio $\gamma _{\text {start}}$

In addition, we further analyze the sensitivity of the initial pruning ratio $\gamma _{\text {start}}$. Since the proposed method adopts an adaptive progressive pruning mechanism, $\gamma _{\text {start}}$ only serves as the initial pruning proportion, and the subsequent pruning strength is dynamically adjusted. Therefore, this parameter should not be set too large, as it may excessively remove training samples at the early stage. Based on this consideration, we select $\gamma _{\text {start}} = 0.1, 0.2, 0.3, 0.4$ for evaluation, which cover a reasonable range from relatively conservative to moderate initial pruning strength. All experiments are conducted on CIFAR-100 under the $p=10$ incremental setting, with all other configurations kept unchanged.

**Table 7:** Accuracy under different values of $\gamma _{\text {start}}$.

$\gamma _{\text {start}}$	0.1	0.2	0.3	0.4
Accuracy (%)	58.90	59.13	58.83	59.07

As shown in Table 7, the performance remains stable within a reasonable range of $\gamma _{\text {start}}$, with an overall variation of less than 0.3 percentage points. Overall, adjusting the initial pruning ratio within a reasonable range does not lead to drastic performance fluctuations, indicating that the proposed method exhibits good stability with respect to this hyperparameter. Moreover, due to the adaptive adjustment in the subsequent pruning process, this parameter does not require delicate tuning to achieve stable performance.

## Conclusion

Existing data pruning methods are mainly designed for static learning scenarios and usually rely on fixed pruning rates or one-time sample scoring schemes. This makes them unsuitable for IL, where data distributions and model states continuously evolve. To address this limitation, we propose PADP, a progressive adaptive data pruning method that enables efficient data selection and training in IL tasks. The method evaluates the importance of each sample by considering both its difficulty and dynamic changes during training, and performs progressive adaptive pruning throughout the training process. This enables the model to balance performance and computational efficiency without relying on preset pruning rates. Extensive experiments show that, compared with existing data pruning methods, PADP achieves improvements in both performance and efficiency; furthermore, its consistent effectiveness across multiple IL frameworks and datasets further validates its generality and robustness. We note that current evaluations are limited to image classification and moderate task sequences. Extending PADP to long task horizons, large-scale datasets, and non-vision domains remains an important direction for future work.

## Data Availability

The datasets used in this study are publicly available. CIFAR-100 is available at https://www.cs.toronto.edu/$\sim$kriz/cifar.html, and Tiny ImageNet is available at https://www.kaggle.com/c/tiny-imagenet. The source code used for all experiments is publicly available at: https://github.com/duanbiqing/PADP.

## References

[1] Shen, L. et al. On efficient training of large-scale deep learning models: A literature review. arXiv:2304.03589 (2023).

[2] Liu, D., Kong, H., Luo, X., Liu, W. & Subramaniam, R. Bringing AI to edge: From deep learning’s perspective. *Neurocomputing* **485**, 297–320 (2022).

[3] Luo, X. et al. Efficient deep learning infrastructures for embedded computing systems: A comprehensive survey and future envision. *ACM Trans. Embedd. Comput. Syst.* **24**, 1–100 (2024).

[4] Hestness, J., Ardalani, N. & Diamos, G. Beyond human-level accuracy: Computational challenges in deep learning. In *Proceedings of the 24th Symposium on Principles and Practice of Parallel Programming*. 1–14 (2019).

[5] Liao, Y. et al. Accelerating federated learning with data and model parallelism in edge computing. *IEEE/ACM Trans. Netw.* **32**, 904–918 (2023).

[6] Shih, A., Belkhale, S., Ermon, S., Sadigh, D. & Anari, N. Parallel sampling of diffusion models. *Adv. Neural Inf. Process. Syst.* **36**, 4263–4276 (2023).

[7] He, Y. et al. Amc: Automl for model compression and acceleration on mobile devices. In *Proceedings of the European Conference on Computer Vision (ECCV)*. 784–800 (2018).

[8] Fan, C., Guo, D., Wang, Z. & Wang, M. Multi-objective convex quantization for efficient model compression. In *IEEE Transactions on Pattern Analysis and Machine Intelligence* (2024). 10.1109/TPAMI.2024.3521589 40030682

[9] Paul, M., Ganguli, S. & Dziugaite, G. K. Deep learning on a data diet: Finding important examples early in training. *Adv. Neural Inf. Process. Syst.* **34**, 20596–20607 (2021).

[10] Guo, C., Zhao, B. & Bai, Y. Deepcore: A comprehensive library for coreset selection in deep learning. In *International Conference on Database and Expert Systems Applications*. 181–195 (Springer, 2022).

[11] Sener, O. & Savarese, S. Active learning for convolutional neural networks: A core-set approach. arXiv:1708.00489 (2017).

[12] Yang, S. et al. Mind the boundary: Coreset selection via reconstructing the decision boundary. In *Forty-first International Conference on Machine Learning* (2024).

[13] Zheng, H., Liu, R., Lai, F. & Prakash, A. Coverage-centric coreset selection for high pruning rates. arXiv:2210.15809 (2022).

[14] Wan, Z. et al. Contributing dimension structure of deep feature for coreset selection. *Proc. AAAI Conf. Artif. Intell.* **38**, 9080–9088 (2024).

[15] Mirzasoleiman, B., Bilmes, J. & Leskovec, J. Coresets for data-efficient training of machine learning models. In *International Conference on Machine Learning*. 6950–6960 (PMLR, 2020).

[16] Tiwari, R., Killamsetty, K., Iyer, R. & Shenoy, P. GCR: Gradient coreset based replay buffer selection for continual learning. In *Proceedings of the IEEE/CVF Conference on Computer Vision and Pattern Recognition*. 99–108 (2022).

[17] Chang, H.-S., Learned-Miller, E. & McCallum, A. Active bias: Training more accurate neural networks by emphasizing high variance samples. *Adv. Neural Inf. Process. Syst.* **30** (2017).

[18] He, M., Yang, S., Huang, T. & Zhao, B. Large-scale dataset pruning with dynamic uncertainty. In *Proceedings of the IEEE/CVF Conference on Computer Vision and Pattern Recognition*. 7713–7722 (2024).

[19] Toneva, M. et al. An empirical study of example forgetting during deep neural network learning. arXiv:1812.05159 (2018).

[20] Zhang, X., Du, J., Li, Y., Xie, W. & Zhou, J. T. Spanning training progress: Temporal dual-depth scoring (TDDS) for enhanced dataset pruning. In *Proceedings of the IEEE/CVF Conference on Computer Vision and Pattern Recognition*. 26223–26232 (2024).

[21] Yang, S. et al. Dataset pruning: Reducing training data by examining generalization influence. arXiv:2205.09329 (2022). 10.1109/TPAMI.2026.3703395 42295952

[22] Chhabra, A., Li, P., Mohapatra, P. & Liu, H. what data benefits my classifier? Enhancing model performance and interpretability through influence-based data selection. In *The Twelfth International Conference on Learning Representations* (2024).

[23] Yang, S., Yang, H., Guo, S., Shen, F. & Zhao, J. Not all data matters: An end-to-end adaptive dataset pruning framework for enhancing model performance and efficiency. arXiv:2312.05599 (2023).

[24] Huang, W., Zhang, Y., Guo, S., Shang, Y.-M. & Fu, X. Dynimpt: A dynamic data selection method for improving model training efficiency. *IEEE Trans. Knowl. Data Eng.* **37**, 239–252 (2024).

[25] Zhou, D.-W. et al. Class-incremental learning: A survey. In *IEEE Transactions on Pattern Analysis and Machine Intelligence* (2024). 10.1109/TPAMI.2024.3429383 39012754

[26] Zhou, D.-W. et al. Deep class-incremental learning: A survey. arXiv:2302.03648 **1**, 6 (2023).

[27] Coleman, C. et al. Selection via proxy: Efficient data selection for deep learning. arXiv:1906.11829 (2019).

[28] He, Y., Xiao, L. & Zhou, J. T. You only condense once: Two rules for pruning condensed datasets. *Adv. Neural Inf. Process. Syst.* **36**, 39382–39394 (2023).

[29] Kirkpatrick, J. et al. Overcoming catastrophic forgetting in neural networks. *Proc. Natl. Acad. Sci.* **114**, 3521–3526 (2017). 28292907 10.1073/pnas.1611835114 PMC5380101

[30] Li, Z. & Hoiem, D. Learning without forgetting. *IEEE Trans. Pattern Anal. Mach. Intell.* **40**, 2935–2947 (2017). 29990101 10.1109/TPAMI.2017.2773081

[31] Rebuffi, S.-A., Kolesnikov, A., Sperl, G. & Lampert, C. H. icarl: Incremental classifier and representation learning. In *Proceedings of the IEEE Conference on Computer Vision and Pattern Recognition*. 2001–2010 (2017).

[32] Douillard, A., Cord, M., Ollion, C., Robert, T. & Valle, E. Podnet: Pooled outputs distillation for small-tasks incremental learning. In *European Conference on Computer Vision*. 86–102 (Springer, 2020).

[33] Yan, S., Xie, J. & He, X. Der: Dynamically expandable representation for class incremental learning. In *Proceedings of the IEEE/CVF Conference on Computer Vision and Pattern Recognition*. 3014–3023 (2021).

[34] Zhu, K., Zhai, W., Cao, Y., Luo, J. & Zha, Z.-J. Self-sustaining representation expansion for non-exemplar class-incremental learning. In *Proceedings of the IEEE/CVF Conference on Computer Vision and Pattern Recognition*. 9296–9305 (2022).

[35] Zhu, F., Zhang, X.-Y., Wang, C., Yin, F. & Liu, C.-L. Prototype augmentation and self-supervision for incremental learning. In *Proceedings of the IEEE/CVF Conference on Computer Vision and Pattern Recognition*. 5871–5880 (2021).

[36] Wang, F., Zhou, D., Ye, H. & Foster, D. Z. Feature boosting and compression for class-incremental learning. In *ECCV FOSTER* (2022).

[37] Zhou, D.-W., Wang, Q.-W., Ye, H.-J. & Zhan, D.-C. A model or 603 exemplars: Towards memory-efficient class-incremental learning. arXiv:2205.13218 (2022).

[38] Zhou, D.-W., Cai, Z.-W., Ye, H.-J., Zhan, D.-C. & Liu, Z. Revisiting class-incremental learning with pre-trained models: Generalizability and adaptivity are all you need. *Int. J. Comput. Vis.* **133**, 1012–1032 (2025).

[39] Zheng, B., Zhou, D.-W., Ye, H.-J. & Zhan, D.-C. Task-agnostic guided feature expansion for class-incremental learning. In *Proceedings of the Computer Vision and Pattern Recognition Conference*. 10099–10109 (2025).

[40] Duan, B. et al. Lodap: On-device incremental learning via lightweight operations and data pruning. arXiv:2504.19638 (2025).

[41] Feldman, V. & Zhang, C. What neural networks memorize and why: Discovering the long tail via influence estimation. *Adv. Neural Inf. Process. Syst.* **33**, 2881–2891 (2020).

[42] Hu, Q., Gao, Y. & Cao, B. Curiosity-driven class-incremental learning via adaptive sample selection. *IEEE Trans. Circuits Syst. Video Technol.* **32**, 8660–8673 (2022).

[43] Acharya, A., Yu, D., Yu, Q. & Liu, X. Balancing feature similarity and label variability for optimal size-aware one-shot subset selection. In *Forty-first International Conference on Machine Learning* (2024).

[44] Raju, R. S., Daruwalla, K. & Lipasti, M. Accelerating deep learning with dynamic data pruning. arXiv:2111.12621 (2021).

[45] Dhar, S. et al. A survey of on-device machine learning: An algorithms and learning theory perspective. *ACM Trans. Internet Things* **2**, 1–49 (2021).

[46] Krizhevsky, A. & Hinton, G. *Technical Report* (University of Toronto, 2009).

[47] Le, Y. & Yang, X. Tiny ImageNet visual recognition challenge. *CS* **231N**(7), 3 (2015).

[48] Xia, X. et al. Moderate coreset: A universal method of data selection for real-world data-efficient deep learning. In *The Eleventh International Conference on Learning Representations* (2022).

[49] Pleiss, G., Zhang, T., Elenberg, E. & Weinberger, K. Q. Identifying mislabeled data using the area under the margin ranking. *Adv. Neural Inf. Process. Syst.* **33**, 17044–17056 (2020).

[50] Tan, H. et al. Data pruning via moving-one-sample-out. *Adv. Neural Inf. Process. Syst.* **36**, 18251–18262 (2023).

[51] Paszke, A. et al. Pytorch: An imperative style, high-performance deep learning library. *Adv. Neural Inf. Process. Syst.* **32** (2019).

[52] He, K., Zhang, X., Ren, S. & Sun, J. Deep residual learning for image recognition. In *Proceedings of the IEEE Conference on Computer Vision and Pattern Recognition*. 770–778 (2016).

